# Supplementary figures and images for: Blockchain for genomics and healthcare: a literature review, current status, classification and open issues
Source: PeerJ. 2021 Sep 30;9:e12130. doi: 10.7717/peerj.12130 (PMC8487622; doi:10.7717/peerj.12130)

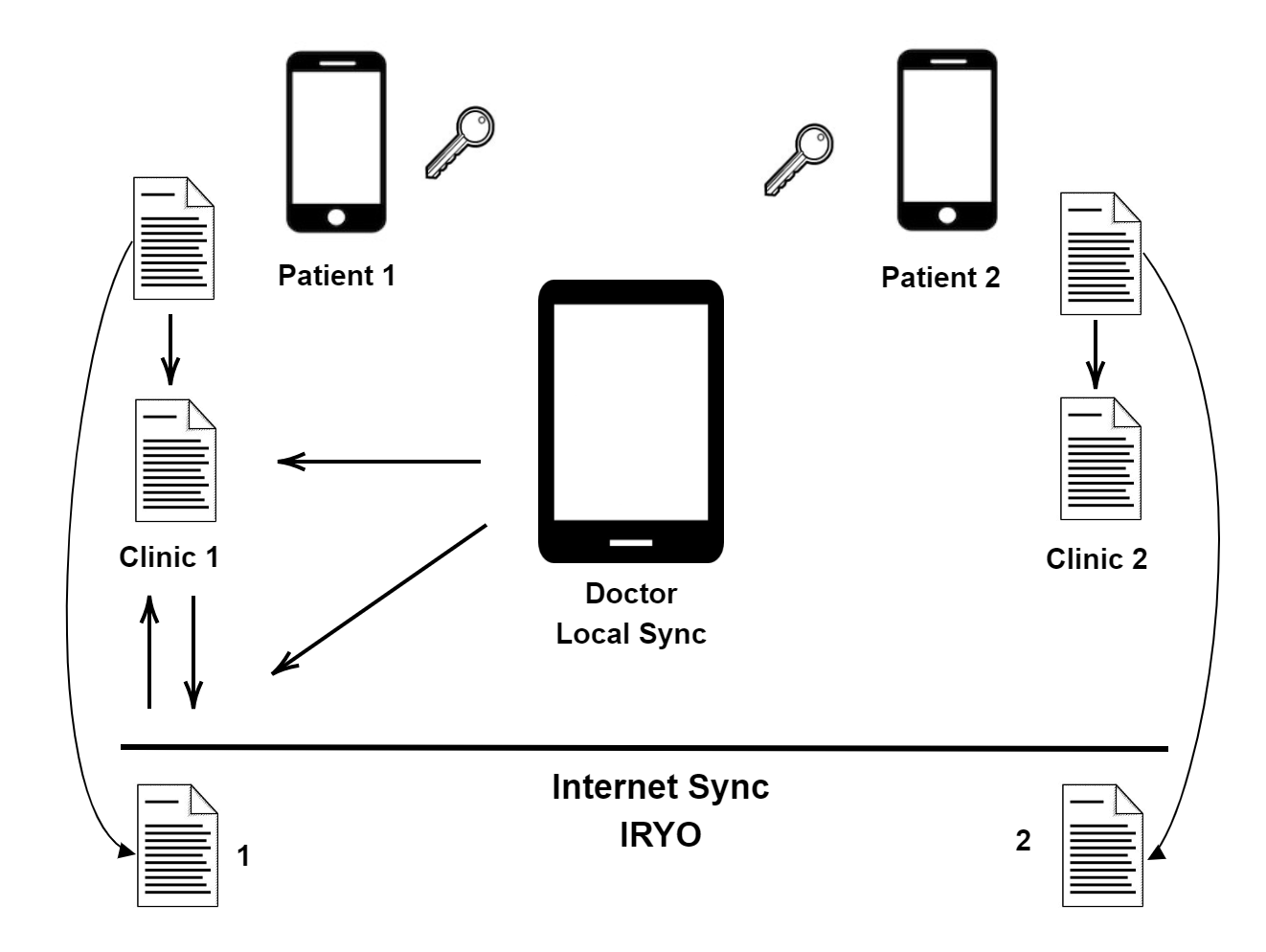

Supplement: Supplemental Information 1 [file peerj-09-12130-s001.png]

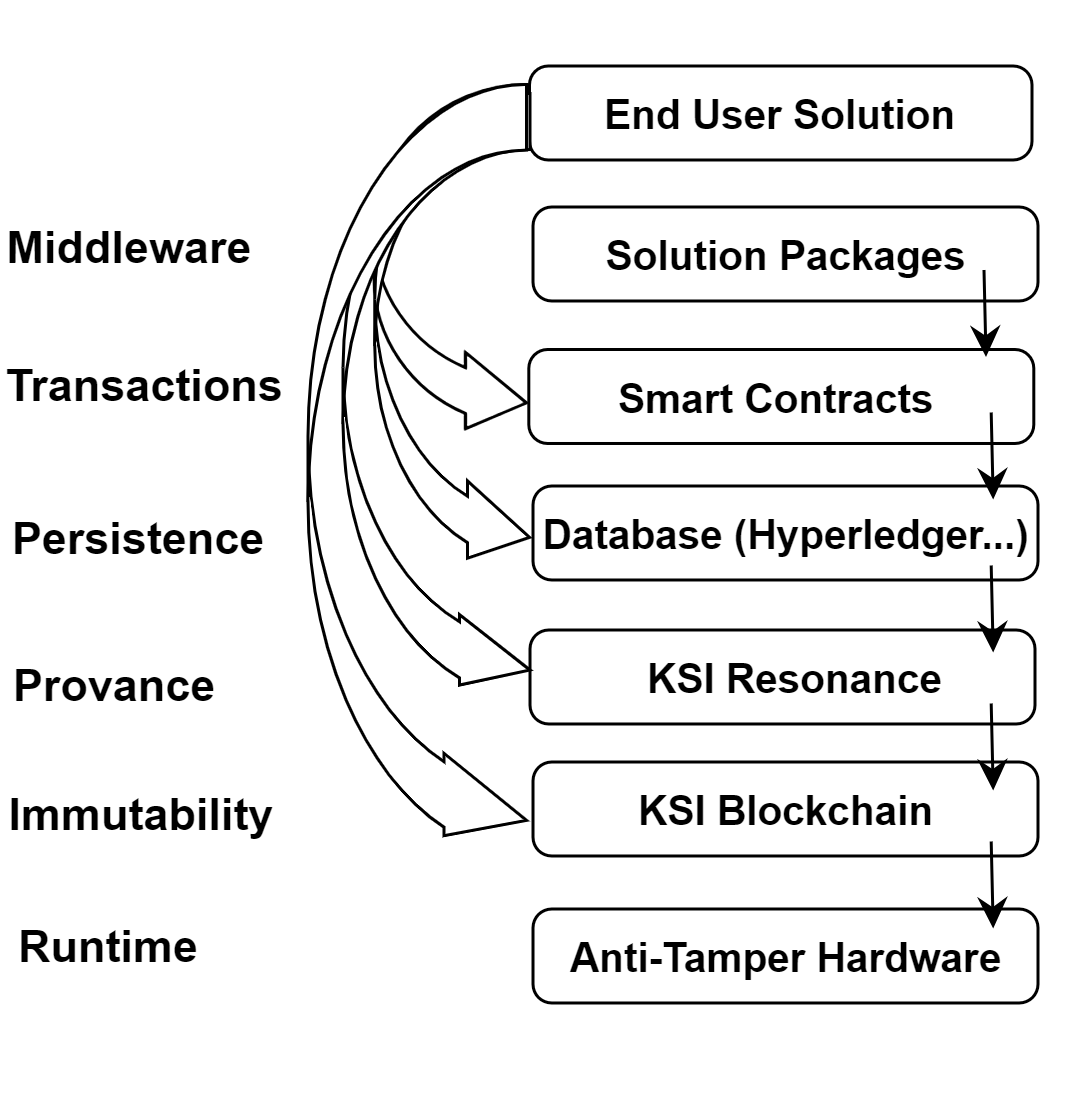

Supplement: Supplemental Information 2 [file peerj-09-12130-s002.png]
